# Thoracic surgeons’ practice and attitude towards surgical antimicrobial prophylaxis in VATS lung surgery: A survey within a large medical consortium

**DOI:** 10.1371/journal.pone.0339389

**Published:** 2026-02-02

**Authors:** Xiaotong Gu, Yue Liu, Yanguo Liu, Jing Huang, Yi Liu, Lin Huang, Xiaohong Zhang, Rongrong Fan

**Affiliations:** 1 Department of Pharmacy, Peking University People’s Hospital, Beijing, China; 2 Department of Pharmacy, China Aerospace Science & Industry Corporation 731 Hospital, Beijing, China; 3 Department of Thoracic surgery, Peking University People’s Hospital, Beijing, China; European Institute of Oncology: Istituto Europeo di Oncologia, ITALY

## Abstract

**Background and objectives:**

Surgical site infections (SSIs) are a significant post-surgery complication, impacting mortality, morbidity, and healthcare costs. Surgical antimicrobial prophylaxis (SAP) is pivotal in SSIs prevention. This study aimed to evaluate the current use of SAP in video-assisted thoracoscopic surgery (VATS) lung surgery in China.

**Methods:**

A descriptive, cross-sectional survey study was conducted among thoracic surgeons within a large medical consortium in order to assess their practice and attitude about SAP. A three-section multiple-choice online questionnaire was designed and distributed via WeChat software to thoracic surgeons. The surgeons’ answers were considered consistent when they were in accordance to clinical guidelines.

**Results:**

89 thoracic surgeons were requested to participate in this study and their response rate was 73.03%. Preoperatively, 60.00% administered antimicrobials, predominantly within 0.5 to 1 hour before surgery, with cefuroxime as the preferred agent. Intraoperatively, 32.31% did not administer additional antimicrobials, and postoperatively, 90.77% prescribed them, often continuing until drainage tube removal. Surgeons frequently upgraded prophylaxis, especially postoperatively. Deviations from guidelines were common, particularly in postoperative SAP duration (76.92%), intraoperative redosing decisions (58.33%), and preoperative SAP administration (40.00%). Departmental habits significantly influenced SAP practices. The primary reason for inconsistencies was the absence of patient-specific considerations in the guidelines, affecting nearly half of the cases. Experienced surgeons were more likely to cite this lack of patient-specific attention as a reason for deviation.

**Conclusion:**

The study underscores the need for updated, multidisciplinary guidelines for VATS lung surgery, emphasizing the importance of a collaborative approach among healthcare professionals to optimize individualized SAP.

## Introduction

Lung cancer was the most commonly diagnosed cancer in 2022, accounting for nearly 2.5 million new cases, or one in eight cancer cases worldwide (12.4% of all cancer cases globally) [1]. Moreover, it was the leading cause of cancer-related deaths, with an estimated 1.8 million fatalities (18.7%) [1]. Thoracic surgery plays a crucial role in the curative treatment of early-stage lung cancer patients by removing thoracic tumors [2]. With significant advancements in thoracic surgery techniques, video-assisted thoracoscopic surgery (VATS) has shown enormous superiority in the field of thoracic surgical treatment and management across various countries [3,4]. It has seen substantial growth and has become the standard of care for lung cancer surgery [4].

Surgical site infections (SSIs) remain a common problem, posing threats to both patient health and healthcare systems [5,6]. International and national clinical practice guidelines, including strategies for surgical antimicrobial prophylaxis (SAP), have been pivotal in establishing optimal protocols aimed at minimizing the incidence of postoperative infections [7–9]. Nevertheless, a single-center study found that postoperative infections persist as a frequent complication following VATS procedures, with an incidence rate of approximately 4.6% to 21.6% [10]. The most frequently reported infections include surgical wound infection (1.9–12.5%), pneumonia (2.5–22.5%), and empyema (0.4–10.0%) [10]. Studies have indicated that poor adherence to guidelines is correlated with higher rates of SSIs [11–13]. Currently, healthcare organizations are focusing on external interventions such as antimicrobial stewardship programs and educational initiatives [14,15]. However, there is a tendency to overlook the intrinsic variability in surgical practices. Antimicrobial prescribing patterns continue to be influenced by the preferences of individual thoracic surgeons, revealing a significant gap in standardized practices [16,17]. This situation underscores the need for a comprehensive investigation into antimicrobial usage patterns during VATS lung surgery, along with a critical evaluation of thoracic surgeons’ perspectives on SAP and their compliance with established guidelines. Baniasadi et al. assessed the knowledge, attitude, and practice of thoracic surgeons regarding SAP [18]. However, since this study was conducted in Iran, its findings might be influenced by local cultural and health system factors. Additionally, the research focused on general thoracic surgery rather than VATS-specific practices. Although the Iranian study evaluated surgeons’ adherence to ASHP guidelines, it did not provide an in-depth analysis of the specific barriers or motivators affecting adherence within the context of VATS lung surgery.

To our knowledge, this is the first study to investigate the practice and attitude of thoracic surgeons towards SAP in VATS lung surgery in China. The primary aim of this study is to elucidate the underlying reasons contributing to the divergence between SAP practices and recommendations set forth by established guidelines. Additionally, we seek to identify prevalent challenges and potential areas for improvement in SAP practice during VATS lung surgery.

## Methods

### Ethical approval and consent to participate

All activities for these investigations were approved by the Ethics Review Board of Peking University People’s Hospital (IRB number 2023PHB294−001) and were conducted in accordance with the Declaration of Helsinki. All participants voluntarily completed the anonymous questionnaire without any financial compensation.

### Questionnaire

To investigate the practice and attitude of thoracic surgeons regarding SAP in VATS lung surgery, a multidisciplinary team comprised of thoracic surgeons, nurses, and pharmacists designed a validated online questionnaire. The questionnaire was designed based on their clinical and scientific expertise, as well as recommendations from established guidelines. To ensure clarity and relevance, a preliminary version was piloted with a small group of thoracic surgeons. Their feedback was instrumental in refining the questions. The final questionnaire (S1 File) consists of 23 questions that are divided into three sections: (a) General information about the participants (questions 1–4); (b)Thoracic surgeons’ practice towards SAP in VATS lung surgery, encompassing preoperative, intraoperative, and postoperative phases, and their adherence to guideline recommendations (questions 5–20); (c) Thoracic surgeons’ attitude towards SAP (questions 21–23). Except for questions 1–6 and 15–16, which are open-ended, all other questions are multiple-choice, with the option for respondents to specify their own responses if they select “Others”.

### Study design

This study was a multicenter, cross-sectional investigation reported in accordance with the Strengthening the Reporting of observational studies in Epidemiology (STROBE) checklist [19]. The target population consisted of thoracic surgeons from a large medical consortium, encompassing one central facility (Department of Thoracic Surgery, Peking University People’s Hospital with an annual surgical volume exceeding 5,000) and seven subcenters spread across four provinces in China (Beijing 4, Shandong 1, Hebei 1, Fujian 1). Online questionnaires, created using the Questionnaire Star tool were distributed via WeChat software between December 1, 2023 and March 31, 2024.

Informed consent was obtained electronically from all participants. Prior to accessing the questionnaire, participants were presented with a detailed information outlining the study purpose, procedures, confidentiality measures, and their right to voluntary participation. They were informed that completion and submission of the questionnaire would be taken as their consent to participate in the study.

### Statistical analysis

Data from the received questionnaires were collected, collated, and statistically analyzed using the Statistical Package for Social Sciences^®^ (SPSS), version 29. Responses were presented as either the absolute number of participants or as percentages. The percentages were calculated based on the number of respondents to each question. Numerical variables were initially assessed using Chi-square test. A *P*-value of less than 0.05 was considered to indicate statistical significance.

## Results

### Demographics and characteristics

Of 89 surgeons, 65 responded, representing a response rate of 73.03%. As seen in Table 1, the job positions range from chief physicians to postgraduates, indicating a broad spectrum of professional levels within the field of thoracic surgery. The majority are affiliated with Grade-A tertiary hospitals (78.46%). Regarding work experience, the largest group is composed of those with <5 years (32.21%), followed by those with 10–20 years of experience (29.23%). Nearly half of the participants (46.15%) reported that their departments manage an average of over 2,000 VATS cases annually.

**Table 1:** Demographics and characteristics of respondents.

Characteristics	No. (%) of respondents (n = 65)
Job position	
Chief physician	8 (12.31%)
Associate chief physician	17 (26.15%)
Attending physician	14 (21.54%)
Resident physician	11 (16.92%)
Postgraduate	15 (23.08%)
Type of hospital	
Grade-A tertiary hospital	51 (78.46%)
Grade-B tertiary hospital	14 (16.92%)
Years of practice (Experience in thoracic surgery)	
< 5	21 (32.21%)
5-10	16 (24.62%)
10-20	19 (29.23%)
> 20	9 (13.85%)
Annual thoracic surgery volume	
< 500	12 (18.46%)
500-1000	11 (16.92%)
1000-2000	12 (18.46%)
> 2000	30 (46.15%)

### The practice of thoracic surgeons about SAP in VATS lung surgery

1. Preoperative use

**Fig 1:**
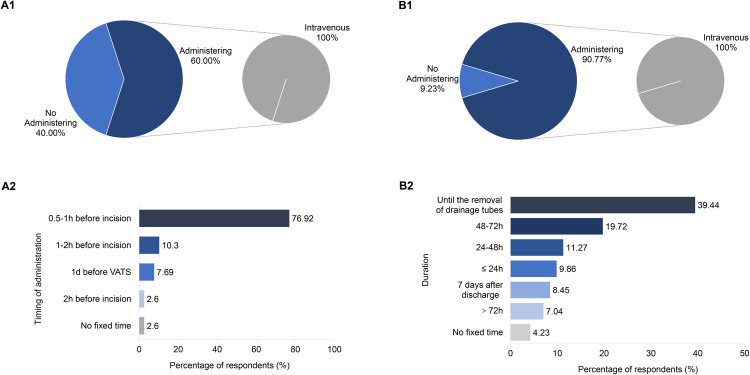
The practice of thoracic surgeons in preoperative and postoperative SAP for VATS lung surgery. A1) Selection of preoperative antimicrobials; A2) Timing of preoperative antimicrobial administration; B1) Selection of postoperative antimicrobials; B2) Duration of postoperative antimicrobial therapy. VATS: video-assisted thoracoscopic surgery.

In the survey, 39 participants (60.00%) affirmed that they administer preoperative antimicrobials for VATS lung surgery. However, 26 participants (40.00%) reported not using preoperative antimicrobials. Among those who advocate for preoperative antimicrobials, 76.92% believe antimicrobials should be administered 0.5 to 1 hour before surgery. The remaining 23.08% administer them outside this window, indicating varied clinical approaches. All participants administer the prophylaxis intravenously, as shown in Fig 1 (A1 and A2).

**Fig 2:**
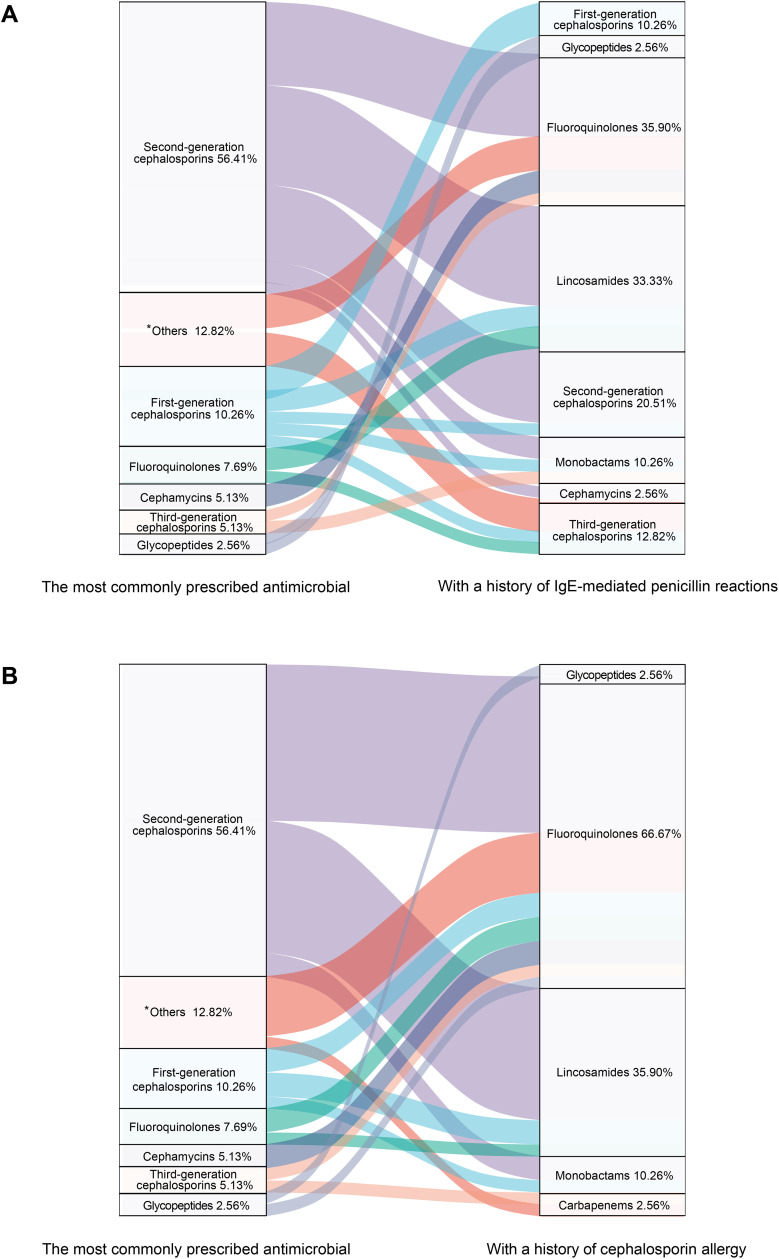
Preferred antimicrobials (without known allergies) and alternatives for known allergies in preoperative SAP for VATS lung surgery. A) preferred substitute for patients with a history of IgE-mediated reaction to penicillin; B) preferred substitute for patients with a history of allergy to cephalosporins. *Others: Piperacillin/Tazobactam or Piperacillin/Sulbactam (10.26%); Unknown (2.56%).

Fig 2 illustrates that cefuroxime, a second-generation cephalosporin, was the preferred choice for preoperative antimicrobials in VATS lung surgery, selected by 56.41% of respondents. Other agents were selected by 12.82% (among which piperacillin & tazobactam/sulbactam accounted for 10.26%), followed by first-generation cephalosporins (cefazolin, 10.26%), and fluoroquinolones (7.69%). For patients with a history of severe IgE-mediated penicillin reactions, fluoroquinolones were the preferred substitute (35.90%), closely followed by clindamycin (33.33%), with cefuroxime still considered by 20.51%. For patients with a history of cephalosporin allergy, fluoroquinolones (66.67%), clindamycin (35.90%), and aztreonam (10.26%) were the most prescribed.

**Fig 3:**
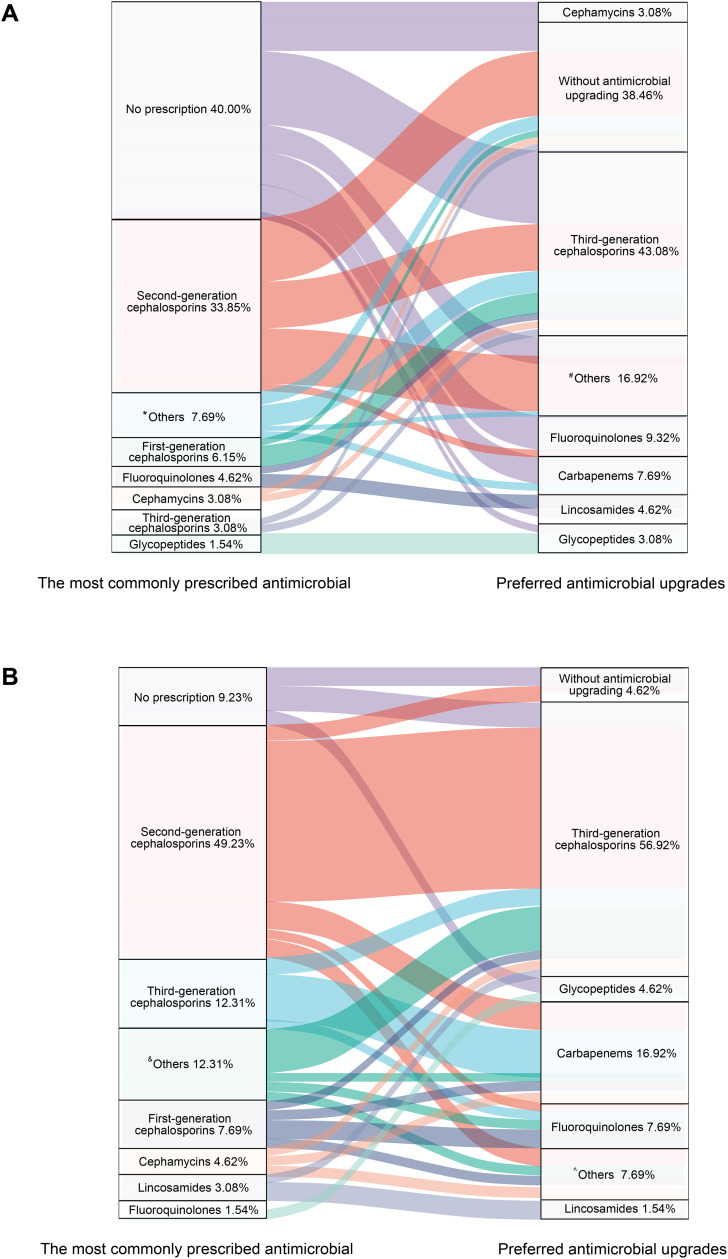
Preferred antimicrobial upgrades for VATS lung surgery. **A)** Preoperative antimicrobial upgrades; **B)** Postoperative antimicrobial upgrades. ^*^Others: Piperacillin/Tazobactam or Piperacillin/Sulbactam (6.15%); Unknown (1.53%); ^#^Others: Piperacillin/Tazobactam or Piperacillin/Sulbactam (13.84%); Unknown (3.08%); ^&^Others: Piperacillin/Tazobactam or Piperacillin/Sulbactam (6.15%); Unknown (6.15%); ^^^Others: Piperacillin/Tazobactam or Piperacillin/Sulbactam (4.61%); Unknown (3.08%).

As shown in Table 2, 38.46% of respondents do not upgrade preoperative antimicrobials. The main reasons for upgrading were immunodeficiency or malnutrition (44.62%), surgical duration exceeding 3 hours (35.38%), and poor glycemic control in diabetics (32.31%). For upgrading preoperative prophylaxis in VATS lung surgery (Fig 3A), third-generation cephalosporins were the most favored (43.08%), followed by others (among which piperacillin & tazobactam/sulbactam accounted for 13.84%), fluoroquinolones (9.32%), and carbapenems (7.69%).

**Table 2:** The situation of upgrading preoperative antimicrobial prophylaxis for VATS lung surgery.

Characteristics	No. (%) of respondents
Immunodeficiency or malnutrition	29 (44.62%)
No upgrade	25 (38.46%)
Estimated surgical duration ＞ 3h	23 (35.38%)
Poor glycemic control in diabetes	21 (32.31%)
Operation types (wedge resection/ segmentectomy/ lobectomy/ sleeve resection/pneumonectomy)	16 (24.62%)
Smoking	16 (24.62%)
Recent use of antimicrobial therapy	15 (23.08%)
Undergoing treatment for malignant tumor (radiotherapy/chemotherapy)	13 (20.00%)
Age > 70 years	12 (18.46%)
Obesity	9 (13.85%)
Extended preoperative hospital stay	8 (12.31%)
Others	2 (3.08%)

1. Intraoperative use

Studies have noted that many surgeons will reflexively prescribe intraoperative antimicrobials [20]. Consistent with these findings, our data presented in Table 3 reveals considerable variation in intraoperative prophylaxis practices among thoracic surgeons. Specifically, 32.31% of surgeons do not administer additional antimicrobials intraoperatively. However, for surgeries exceeding 3 hours (50.77%) or 4 hours (15.38%), some surgeons choose to repeat antimicrobials to ensure adequate prophylaxis. Likewise, significant intraoperative bleeding, exceeding 1000 mL (32.31%) or 1500 mL (10.77%), also leads to the administration of additional antimicrobials.

**Table 3:** The practice of intraoperative antimicrobial prophylaxis for VATS lung surgery.

Characteristics	No. (%) of respondents
Practice of repeating antimicrobials	
Without additional antimicrobials	21 (32.31%)
Surgical duration ＞ 3h	33 (50.77%)
Surgical duration ＞ 4h	10 (15.38%)
Intraoperative bleeding volume＞1000 mL	21 (32.31%)
Intraoperative bleeding volume＞1500 mL	7 (10.77%)
Others	1 (1.54%)
Preferred repeating antimicrobials	
Repeating preoperative antimicrobials	35 (72.92%)
Third-generation cephalosporins	8 (16.66%)
Second-generation cephalosporins	4 (8.33%)
First-generation cephalosporins	3 (6.25%)
Fluoroquinolones	2 (4.17%)
Lincosamides	2 (4.17%)
Carbapenems	2 (4.17%)

In terms of preferred antimicrobials for repetition, 72.92% of surgeons prefer to continue with the preoperative antimicrobials, suggesting a trend toward consistent prophylactic coverage. Nevertheless, alternative choices include third-generation cephalosporins (16.66%), second-generation cephalosporins (cefuroxime, 8.33%) and first-generation cephalosporins (cefazolin, 6.25%).

1. Postoperative use

Almost all respondents (90.77%) administer postoperative antimicrobials for VATS lung surgery, while 9.23% do not. Among those who prescribed antimicrobials, all prefer intravenous administration. The duration of antimicrobial administration varies considerably among thoracic surgeons. Specifically, 39.44% continue until the removal of drainage tubes, 19.72% administer for 48–72 hours, and 11.27% administer for 24–48 hours (Fig.1 (B1&B2)).

As can be seen in Fig 3B, postoperative antimicrobials selection reveals that cefuroxime is the top choice for nearly half of the respondents (49.23%), followed by third-generation cephalosporins (12.31%) and first-generation cephalosporins (cefazolin, 7.69%).

The situation regarding the upgrading of postoperative antimicrobial prophylaxis for VATS lung surgery is presented in Table 4. The upgrading is primarily based on clinical manifestations (87.69%), imaging findings of infection (72.13%), and microorganism detection (66.15%). Other factors include poor sputum production (47.69%), respiratory symptoms (46.15%), abnormal WBC counts (43.08%), mechanical ventilation duration (29.23%), and significant CRP increase (26.15%). Operation type influences 20% of decisions, while only 4.62% reported no upgrading. Third-generation cephalosporins are the most frequently chosen option for upgrading (56.92%). Carbapenems were the second most common choice (16.92%), followed by fluoroquinolones (7.69%).

**Table 4:** The situation of upgrading postoperative antimicrobial prophylaxis for VATS lung surgery.

Characteristics	No. (%) of respondents
Clinical manifestations, such as elevated body temperature or postoperative purulent airway secretions, etc.	57 (87.69%)
Imaging that reveals signs of infection	47 (72.13%)
Detection of pathogenic microorganisms	43 (66.15%)
Poor postoperative sputum production	31 (47.69%)
Newly appeared cough after surgery, or worsening of existing cough or respiratory symptoms	30 (46.15%)
WBC > 12 × 10^9^/L or < 4 × 10^9^/L	28 (43.08%)
Duration of invasive mechanical ventilation	19 (29.23%)
Significant increase in CRP compared to preoperative levels	17 (26.15%)
Operation types (wedge resection/ segmentectomy/ lobectomy/ sleeve resection/pneumonectomy)	13 (20%)
No upgrade	3 (4.62%)
Others	1 (1.54%)

1. Comparison of consistency between SAP practices of the respondents and the guidelines

**Fig 4:**
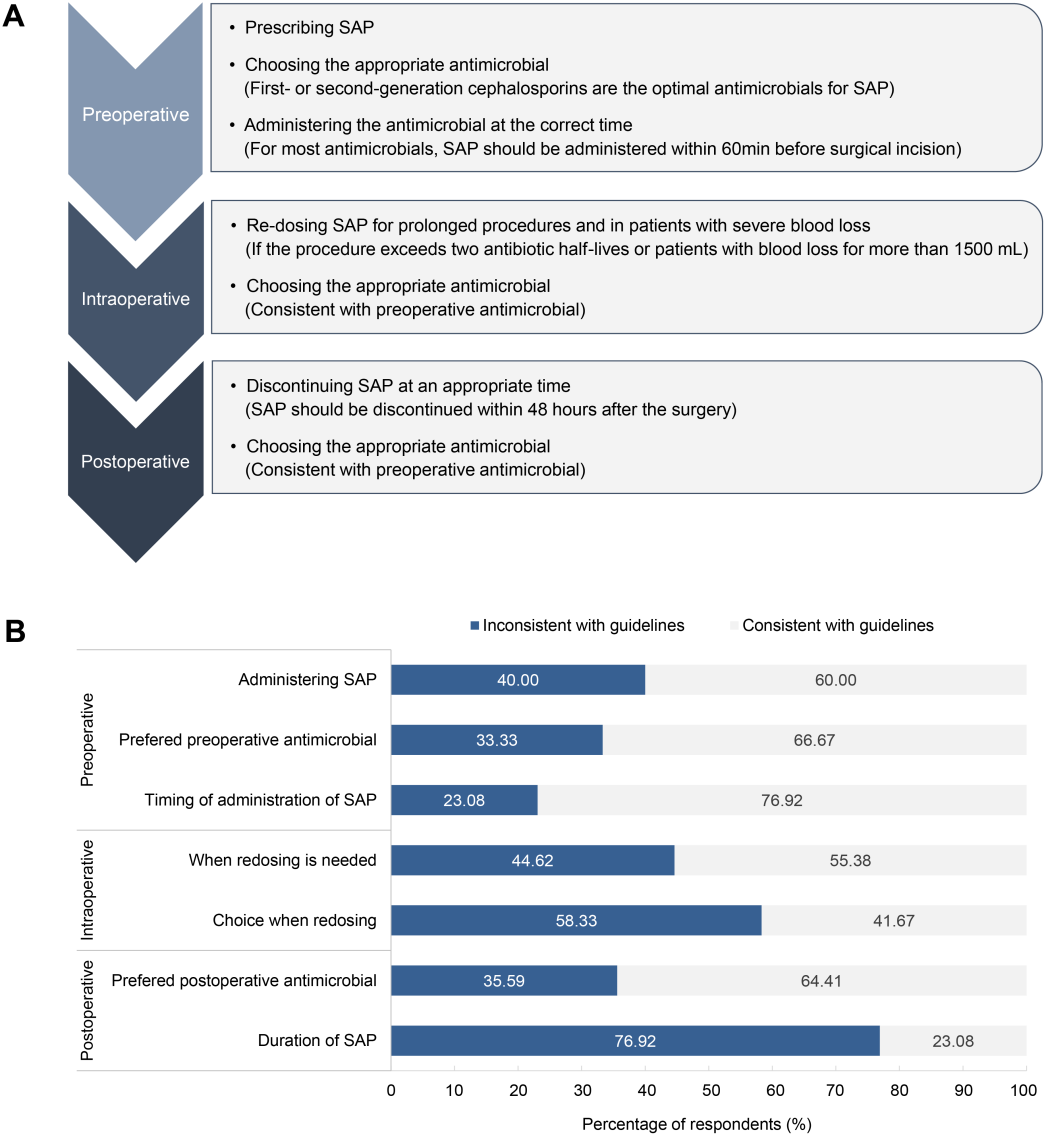
SSIs prevention guidelines suggestions for SAP and comparison of consistency between prescription practices and guidelines. **A)** Recommendations for SAP from SSIs prevention guidelines; **B)** Percentage of compliance with guidelines. SAP: surgical antimicrobial prophylaxis.

Fig 4 highlights recommendations for SAP from SSIs prevention guidelines, the recommendations are consistent between international and Chinese guidelines [7–9]. Our study reveals significant discrepancies between guidelines and clinical practices, especially in the postoperative phase. In the postoperative setting, the duration of SAP administration deviated from guidelines in 76.92% of cases. Intraoperatively, the choice of redosing agent was inconsistent in 58.33% of cases, while the determination of when redosing is needed was inconsistent in 44.62% of cases. In the preoperative phase, the decision on whether to administer antimicrobial agents had a 40.00% discrepancy rate compared to guidelines, the choice of preferred antimicrobials deviated from the guidelines in 33.33% of cases, while the inconsistency in the timing of SAP administration was the lowest, at 23.08%.

### The attitude of thoracic surgeons about SAP in VATS lung surgery

Among 65 respondents, a variety of reasons were given for SAP both in preoperative and postoperative settings, as shown in Table 5. Departmental habits emerged as a significant factor, accounting for 36.92% and 67.69% of the surgeons’ preoperative and postoperative SAP practices, respectively. Standard guidelines and hospital regulations also played a significant role. The most commonly cited reason for deviations from guidelines was the perceived lack of patient-specific considerations, accounting for nearly half of the surgeons’ responses (47.69%). Some surgeons believe that the risk of postoperative infection is low when using drugs based on experience (13.84%) and the infectious risk may increase when following the guidelines (12.31%).

**Table 5:** The attitude of thoracic surgeons towards SAP in VATS lung surgery.

	Total No. (%) n = 65	Years of practice <10, No. (%) n = 37	Years of practice ≥10, No. (%) n = 28	*P* value
Reasons for preoperative SAP practices				
Departmental habits	24 (36.92%)	13 (35.13%)	11 (39.29%)	0.798
Standard guidelines	19 (29.23%)	7 (18.92%)	12 (42.86%)	0.054
Hospital regulations	17 (26.15%)	7 (18.92%)	10 (35.71%)	0.108
Personal experience	14 (21.54%)	6 (16.22%)	8 (28.58%)	0.361
Instructions from superiors	10 (15.38%)	7 (18.92%)	3 (10.71%)	0.495
Pharmacist’s suggestion	9 (13.85%)	5 (13.51%)	4 (14.29%)	1
Reasons for postoperative SAP practices				
Departmental habits	44 (67.69%)	27 (72.97%)	17 (60.71%)	0.422
Standard guidelines	36 (55.38%)	20 (54.05%)	16 (57.14%)	1
Hospital regulations	30 (46.15%)	17 (45.95%)	13 (46.43%)	1
Personal experience	28 (43.08%)	15 (40.54%)	13 (46.43%)	0.801
Instructions from superiors	20 (30.77%)	14 (37.83%)	6 (21.43%)	0.184
Pharmacist’s suggestion	13 (20.00%)	8 (21.62%)	5 (17.86%)	0.764
The main cause of the deviations between the SAP practices and standard guidelines				
The guideline does not consider patient-specific differences	31 (47.69%)	13 (35.14%)	18 (64.29%)	**0.026**
Strict adherence to the guideline	11 (16.92%)	8 (21.62%)	3 (10.71%)	0.326
The risk of postoperative infection is low when using antimicrobials are used based on experience	9 (13.84%)	6 (16.22%)	3 (10.71%)	0.721
Administering antimicrobials according to the guideline may increase the risk of postoperative infection	8 (12.31%)	4 (10.81%)	4 (14.29%)	0.717
The guideline lacks sufficient evidence-based medical evidence	4 (6.15%)	4 (10.81%)	0	0.128
Following the instructions of superiors	2 (3.08%)	2 (5.41%)	0	0.320

A chi-square test showed no link between surgical experience and antimicrobial choice or attitudes towards SAP guidelines. However, there was a statistical difference in the reasons for guideline deviations: surgeons with ≥ 10 years of practice were more often cited lack of patient-specific considerations compared to surgeons with < 10 years of practice (64.29% vs. 35.14%, *P* = 0.026). A higher proportion of thoracic surgeons with < 10 years of practice (21.62%) chose strict adherence to the guideline compared to those with ≥ 10 years of experience (10.71%), although the difference was not statistically significance (*P* = 0.326).

## Discussion

This questionnaire-based survey is the first study to evaluate thoracic surgeons’ practice and attitude towards SAP in VATS lung surgery in China. As SAP became evident, antimicrobials are routinely prescribed in surgical procedures yielded [21]. However, the application of antimicrobials as prophylactic agents in surgery affected by multiple and complicated factors [17,22,23]. Our study has showed that SAP in VATS lung surgery exist wide heterogeneity in preoperative, intraoperative, and postoperative settings and deviations from established guidelines. The main cause of inconsistencies in SAP practices, affecting 76.92% of postoperative, 58.33% of intraoperative, and 40% of preoperative cases, was the lack of patient-specific considerations in guidelines, particularly emphasized by surgeons with ≥10 years of experience.

According to the incision classification, VATS for lung diseases is classified as type II, possible contamination incision, and SAP has been widely used to prevent SSIs in clinical practice [24–26]. The optimal solution needs to achieve a balance between efficacy against the likely pathogens that occur in the surgical site and minimizing the risk of adverse effects. Besides, the optimal solution must be cheap, higher sensitivity, lower toxicity and with a half-life sufficiently prolonged to maintain adequate concentration of drug until the surgical wound has been closed [27–29]. In this survey, 60.00%, 67.69%, and 90.77% respondents prescribe antimicrobial drugs for preoperative, intraoperative, and postoperative use, respectively. The most commonly prescribed antimicrobial is the second generation cephalosporins, cefuroxime, in accordance with guidelines which offer evidence-based recommendations on various aspects of SAP [21]. In fact, the first generation cephalosporins, cephazolin, is also recommended by guidelines as the choice of antimicrobial unless contraindicated [9].The reason for the choice of intravenous cefuroxime may be related to it has a broader spectrum of active coverage against aerobic and anaerobic Gram-positive and Gram-negative bacteria [30]. And the selection of cefuroxime as a preferred antimicrobial of choice by majority of our respondents indicated their relatively good knowledge about this issue. The use of SAP is relatively complicated when patients present a history of severe IgE-mediated penicillin reactions or cephalosporin allergy [18,21,31]. In this study, fluoroquinolones and clindamycin are considered as the alternative solutions, which is similar with other survey [32]. In fact, while the guidelines provide some antimicrobial options for SAP when patients have a history of allergies, they do not offer particularly clear guidance, which is one of the main reasons for the heterogeneity in thoracic surgeons’ choices.

The efficacy of SAP also depends on the timing of administration and the duration of prophylaxis [33]. Our study has revealed significant variability in the timing and duration of antimicrobial prophylaxis among the respondents. Sufficient evidence suggests that administration during 0–60 minutes before skin incision is the optimal time window for SAP when cefuroxime is used as a prophylactic antimicrobial [33–35]. And our study revealed a high adherence rate of surgeons who use preoperative antimicrobial prophylaxis to the timing of SAP administration. In response to specific conditions, such as substantial blood loss and prolonged surgical procedures, intraoperative redosing of antimicrobial prophylaxis has been proposed as an effective measure to reduce the incidence of SSIs[28]. Guidelines recommend redosing antimicrobials in case of blood loss that exceeds 1500 mL or the duration of the operative procedure exceeds two half-lives of the antimicrobial [7–9]. Some of the surgeons surveyed in this study express that repeat intraoperative administration should be used in case of blood loss that exceeds 1000 mL or surgery lasts more than 3 h, but 32.31% have expressed that they never redosing antimicrobial prophylaxis. The non-compliance with the redosing of antimicrobial prophylaxis may be due to the fact that during intraoperative bleeding, thoracic surgeons tend to be more focused on the surgery itself, thus overlooking the need for redosing antimicrobial prophylaxis. However, it is worth noting that a considerable number of surgeons are observed incorrectly administer two or more doses of SAP during surgery [12,36].

Additionally, both international and Chinese guidelines recommend that the duration of SAP is for no more than 24 h or 48h postoperatively [9,35,37,38]. We found that our participants have a tendency to routinely continue antimicrobial prophylaxis until the removal of drainage tubes. In recent years, with the continuous improvement of surgical techniques, the duration of chest tube after lung surgery has been constantly reduced, to approximately1.96–2.85 days [39,40]. This likely aligns with the guideline recommendation of SAP lasting less than 48 hours. Actually, unsuitable use of antimicrobials after surgery is widely observed among surgeons around the world [41–46]. Recent studies demonstrate that postoperative prophylaxis of more than 24 hours is the most common cause of non-compliance [11,47]. The deviation from recommended antimicrobial prophylaxis guidelines presumably because surgeons believe that prolonged antimicrobial administration is safe and more efficient in reducing SSIs incidence [43]. Moreover, in consideration of infection risk factors such as non-sterile environment beyond the control of the surgeons, surgeons will prefer giving prolonged SAP which is under the control to alleviate their fear of unknown [48]. However, prolonged use of antimicrobials after surgery may lead to the emergence of resistant bacteria strain and significant economic burden on healthcare systems [21,33,43]. A latest observational cohort study has also revealed that majority of patients received antimicrobials for prophylaxis after surgery in low- and middle-income countries, which resulting in the length of hospital stay is 1.4 days longer [49]. Meaningly, a study on developed countries found that postoperative use of antimicrobials is relatively low [20]. This difference may also be explained by economic level of the country can affect the antimicrobial prophylaxis practices to some extent.

Furthermore, there are a variety of patient-specific characteristics such as smoking, age, diabetes mellitus, obesity, a weaker immune system or other co-morbidities that may increase the postoperative infective complications and influence pharmacokinetics, pharmacodynamics and clinical efficacy of antimicrobials [35,50]. The majority of respondents thought that antimicrobial prophylaxis should be upgraded when patients face higher risks of postoperative infection. And the most common reasons our respondents prescribed upgrading postoperative antimicrobials are clinical infection symptoms, postoperative radiological features and symptoms and detection of pathogenic microorganisms. In such cases, third-generation cephalosporins is the most common choice among these participants. However, in these cases, whether to upgrade the antimicrobials requires more investigation and evidence-based strategies.

Guidelines for the appropriate use of antimicrobials are designed to provide a standardized approach in thoracic surgeon’s practice [27]. However, our participants show lack of confidence in these existing guidelines which are considered to have not provide enough and generalized evidence-based basis. Based on feedback from our respondents, the selection of appropriate antimicrobials for prophylaxis in VATS lung surgery most easily affected by the departmental habits, followed by guidelines. These participants were concerned about post-VATS infections after using short-term preoperative antimicrobial prophylaxis. They argue that empiric antimicrobial therapy may pose a lower risk of postoperative infection than following guidelines.

Certainly, not all surgical procedures face equal risk of SSIs, and individualized approach is needed to ensure that prophylactic antimicrobial choices align more closely with different surgeries facing the unique infection risks [21]. Unfortunately, there is limited guidance on selecting appropriate SAP in patients with specific physiological and pathological characteristics such as large tumors, diabetes, liver and kidney failure, substantial blood loss, and patients colonized with MDR [28]. Smoking patients, for instance, have more colonization of bacteria in their airway, and guidelines are not clear that whether cefazolin and cefuroxime are still suitable for smoking patients [51]. Additionally, participants have emphasized that different types of surgeries, such as wedge resection, segmentectomy, lobectomy, sleeve resection, and pneumonectomy, can create confusion regarding the selection of prophylactic antimicrobials. The growing popularity of non-intubated thoracic surgery and tubeless video-assisted thoracoscopic surgery underscores the necessity for further research on their impact on perioperative antimicrobial prophylaxis [52,53]. As these techniques become more prevalent, it is essential to understand their implications for antimicrobial use to optimize patient outcomes and reduce the risk of postoperative infections. Therefore, this heterogeneity in decision-making regarding antimicrobial use may stem from confusion over the efficacy of prophylactic regimens in patients with a higher risk of infection, the complexity of the dynamic nature of antimicrobials, encompassing antimicrobial pharmacology and bacterial resistance patterns, as well as the insufficient and inadequate evidence to guide the prophylactic administration of antimicrobials in the local context. Previous study has also reported that surgeons relying on personal knowledge and experience rather than on recommendations of guidelines and formal policy [54]. And locally developed guidelines may be more useful than national ones [55].

Moreover, our research has revealed that as surgeons gain more work experience, they become increasingly focused on patient individualization and place more emphasis on the immediate safety of patients rather than potential hazards such as antimicrobial resistance (AMR) caused by excessive antimicrobials. Actually, most of surgeons who are at the forefront of preventing and managing infections underestimate the problem of AMR and should take a proactive role in ensuring the avoidance of inappropriate and unnecessary antimicrobial use [23]. Thus, scientific and comprehensive guidance and adequate evidence-based recommendations are expected by our participants to meet the needs of individual surgical treatment of patients. A detailed stratification of risk of SSIs for patients which is developed based on different aspects including age, pathophysiological characteristics, surgical type, surgical time, length of hospital stay are much-anticipated, of course corresponding preventive infections practical measures equally expected. It is high time that thoracic surgeons, anesthesiologists, nurses, pharmacists and infectious disease physicians come together to create the evidence for optimal antimicrobial individualized treatment ranging from the correct indication to the right medication, mode of application, dosage and therapy duration in VATS lung surgery. In particular, anesthesiologists, as critical intraoperative team members, are uniquely positioned to monitor surgical duration and blood loss, and to provide timely prompts for antimicrobial redosing, thereby ensuring the real-time and precise execution of SAP protocols.

Some limitations must be acknowledged in this descriptive survey. First, this was a cross-sectional study conducted over a defined period, which captures a snapshot of current practice but cannot assess how SAP practices evolve over time or in response to guideline updates. Second, the survey involved thoracic surgeons primarily from tertiary hospitals in relatively developed provinces of China. The sample size, though collected diligently within a major consortium, remains limited, and the practices may not be fully representative of all regions within China, particularly those with different economic statuses or healthcare resources. Consequently, the generalizability of our findings to other countries or diverse healthcare settings may be limited. Third, approximately 23% of the respondents were postgraduate trainees. Although they participate in surgical care under supervision and their perspectives are valuable for understanding training environments, their lack of independent operating experience may influence the interpretation of certain practice patterns, particularly those requiring seasoned clinical judgment. Fourth, as a multiple-choice survey, it is subject to inherent methodological limitations such as non-responder bias, recall bias, and selection bias.

## Conclusion

This survey presents a lack of acceptance of the current guidelines among thoracic surgeons and highlights significant variations in preoperative, intraoperative and postoperative antimicrobial use for VATS lung surgery within a large medical consortium in China. Recognizing the deviation between SAP practices and the standard guidelines’ recommendations, it may be beneficial to focus future interventions on alleviating surgeons’ concerns about postoperative infection complications. Individualizing SAP for VATS was a primary concern for our participants. Surgeon-specific, surgical, and patient factors that should be considered for the optimal antimicrobial regimen. High-quality, targeted guidelines and randomized studies tailored to lung surgery patients are needed to determine the appropriate SAP, including the correct antimicrobials, timing of administration, optimal dosage, and duration in VATS lung surgery.

## Supporting information

S1 File Questionnaire. (DOCX)
