# Supplementary material for: Thoracic surgeons’ practice and attitude towards surgical antimicrobial prophylaxis in VATS lung surgery: A survey within a large medical consortium
Source: PLoS One. 2026 Feb 2;21(2):e0339389. doi: 10.1371/journal.pone.0339389 (PMC12863502; doi:10.1371/journal.pone.0339389)
Supplement: S1 File — (DOCX) [file pone.0339389.s001.docx]

**Appendix:**

**Questionnaire**

*The purpose of this survey is to* *investigate the practice and attitude of thoracic surgeons about surgical antimicrobial prophylaxis (SAP) in the field of video-assisted thoracoscopic surgery (VATS) lung surgery. Your responses will be assumed to reflect your professional experiences within the one-month period prior to completing the questionnaire.*

*If you decide to participate in this survey, your personal information and questionnaire data will be kept strictly confidential and used for research purposes only. We will make every effort, within the limits permitted by law, to protect your privacy. Your participation in this research is entirely voluntary. You may refuse to participate if you are unwilling.*

*If you have read the above information about this survey, have given it sufficient consideration, and have decided to participate, please proceed with the questionnaire below. This questionnaire composed of a total of 23 questions, which will take you no longer than 8 minutes to complete.* *Your reply will be assumed on completion and submission of the questionnaire. We appreciate your time and contribution.*

**General information**

1. What is your job position?
   1. Chief physician
   2. Associate chief physician
   3. Attending physician
   4. Resident physician
   5. Postgraduate
2. The type of hospital you work in?
   1. Grade-A tertiary hospital
   2. Grade-B tertiary hospital
3. How many years have you been in practice (Experience in thoracic surgery)?
   1. ＜ 5 years
   2. 5-10 years
   3. 10-20 years
   4. > 20 years
4. How many VATS procedures does your department perform each year?
   1. ＜ 500
   2. 500-1000
   3. 1000-2000
   4. >2000

**The practice of thoracic surgeons about SAP in VATS lung surgery**

*Preoperative use*

1. Do you routinely prescribe antimicrobial prophylaxis for VATS lung surgery?
   1. Yes
   2. No
2. If you prescribe antimicrobial, how do you administer them?
   1. Intravenous
   2. Oral
3. When do you routinely initiate preoperative antimicrobial? (Multiple choice)
   1. 1d before VATS
   2. 2h before incision
   3. 1-2h before incision
   4. 0.5-1 h before incision
   5. No fixed time
4. Assuming no patient allergies, what antimicrobial do you generally select for preoperative prophylaxis? (Multiple choice)
   1. Cefazolin
   2. Cefuroxime
   3. Cefoperazone-Sulbactam
   4. Ceftazidime
   5. Ceftriaxone
   6. Cefmetazole
   7. Piperacillin-sulbactam
   8. Piperacillin-tazobactam
   9. Levofloxacin
   10. Moxifloxacin
   11. Ertapenem
   12. Meropenem
   13. Imipenem
   14. Gentamicin
   15. Aztreonam
   16. Clindamycin
   17. Vancomycin
   18. Others, please specify_________
5. For patients with a history of IgE-mediated reaction to penicillin, what’s your preferred agent for SAP? (Multiple choice)
   1. Cefazolin
   2. Cefuroxime
   3. Cefoperazone-Sulbactam
   4. Ceftazidime
   5. Ceftriaxone
   6. Cefmetazole
   7. Levofloxacin
   8. Moxifloxacin
   9. Ertapenem
   10. Meropenem
   11. Imipenem
   12. Gentamicin
   13. Aztreonam
   14. Clindamycin
   15. Vancomycin
   16. Others, please specify _________
6. For patients with a history of cephalosporin allergy, what’s your preferred agent for antimicrobial prophylaxis? (Multiple choice)
   1. Levofloxacin
   2. Moxifloxacin
   3. Ertapenem
   4. Meropenem
   5. Imipenem
   6. Gentamicin
   7. Aztreonam
   8. Clindamycin
   9. Vancomycin
   10. Others, please specify _________
7. Under what circumstances would you choose to upgrade preoperative antimicrobial prophylaxis for VATS lung surgery? (Multiple choice)
   1. No upgrade
   2. Operation types (wedge resection/segmentectomy/ lobectomy/sleeve resection/ pneumonectomy)
   3. Estimated surgical duration ＞3h
   4. Smoking
   5. Obesity
   6. Age ＞ 70 years
   7. Immunodeficiency or malnutrition
   8. Poor glycemic control in diabetes
   9. Undergoing treatment for malignant tumor (radiotherapy/chemotherapy)
   10. Extended preoperative hospital stay
   11. Recent use of antimicrobial therapy
   12. Others, please specify_________
8. What’s your preferred agent for upgrading antimicrobial prophylaxis? (Multiple choice)
   1. Cefoperazone-Sulbactam
   2. Ceftazidime
   3. Ceftriaxone
   4. Cefmetazole
   5. Piperacillin-sulbactam
   6. Piperacillin-tazobactam
   7. Levofloxacin
   8. Moxifloxacin
   9. Ertapenem
   10. Meropenem
   11. Imipenem
   12. Gentamicin
   13. Aztreonam
   14. Clindamycin
   15. Vancomycin
   16. Others, please specify _________

*Intraoperative use*

1. Under what circumstances would you choose to redose antimicrobial intraoperatively for VATS lung surgery? (Multiple choice)
   1. Without additional antimicrobials
   2. Surgical duration＞3h
   3. Surgical duration＞4h
   4. Intraoperative bleeding volume＞1000 mL
   5. Intraoperative bleeding volume＞1500 mL
   6. Others, please specify _________
2. If redosing is required, what’s your preferred antimicrobial for repetition? (Multiple choice)
   1. Repeating preoperative antimicrobials
   2. Cefazolin
   3. Cefuroxime
   4. Cefoperazone-Sulbactam
   5. Ceftazidime
   6. Ceftriaxone
   7. Cefmetazole
   8. Piperacillin-sulbactam
   9. Piperacillin-tazobactam
   10. Levofloxacin
   11. Moxifloxacin
   12. Ertapenem
   13. Meropenem
   14. Imipenem
   15. Gentamicin
   16. Aztreonam
   17. Clindamycin
   18. Vancomycin
   19. Others, please specify _________

*Postoperative use*

1. Do you routinely prescribe postoperative antimicrobial for VATS lung surgery?
   1. Yes
   2. No
2. If you prescribe postoperative antimicrobial, how do you administer them?
   1. Intravenous
   2. Oral
3. What antimicrobial do you generally use as postoperative prophylaxis?(Multiple choice)
   1. Cefazolin
   2. Cefuroxime
   3. Cefoperazone-Sulbactam
   4. Ceftazidime
   5. Ceftriaxone
   6. Cefmetazole
   7. Piperacillin-sulbactam
   8. Piperacillin-tazobactam
   9. Levofloxacin
   10. Moxifloxacin
   11. Ertapenem
   12. Meropenem
   13. Imipenem
   14. Gentamicin
   15. Aztreonam
   16. Clindamycin
   17. Vancomycin
   18. Others, please specify _________
4. Under what circumstances would you choose to upgrade postoperative antimicrobial for VATS lung surgery? (Multiple choice)
   1. No upgrade
   2. Operation types (wedge resection/segmentectomy/ lobectomy/sleeve resection/ pneumonectomy)
   3. Duration of invasive mechanical ventilation
   4. Clinical manifestations, such as elevated body temperature or postoperative purulent airway secretions, etc.
   5. Poor postoperative sputum production
   6. Newly appeared cough after surgery, or worsening of existing cough/respiratory symptoms
   7. WBC>12×10^9^/L or<4×10^9^/L
   8. Significant increase in CRP compared to preoperative
   9. Imaging shows signs of infection
   10. Detection of pathogenic microorganisms
   11. Others, please specify _________
5. If upgrading was necessary, what’s your preferred antimicrobial? (Multiple choice)
   1. Cefoperazone-Sulbactam
   2. Ceftazidime
   3. Ceftriaxone
   4. Cefmetazole
   5. Piperacillin-sulbactam
   6. Piperacillin-tazobactam
   7. Levofloxacin
   8. Moxifloxacin
   9. Ertapenem
   10. Meropenem
   11. Imipenem
   12. Gentamicin
   13. Aztreonam
   14. Clindamycin
   15. Vancomycin
   16. Others, please specify _________
6. If you prescribe postoperative antimicrobial, for how long do you provide prophylaxis? (Multiple choice)
   1. ≤24 hours
   2. 24 – 48 hours
   3. 48 – 72 hours
   4. > 72 hours
   5. Until the removal of drainage tubes
   6. 7 days after discharge
   7. No fixed time

**The attitude of thoracic surgeons about SAP in VATS lung surgery**

1. What is your main consideration factor for choosing the preoperative antimicrobial? (Multiple choice)
   1. National guidelines
   2. Hospital regulations
   3. Departmental habits
   4. Personal experience
   5. Instructions from superiors
   6. Pharmacist's suggestion
   7. Others, please specify _________
2. What is your main consideration factor for choosing the postoperative antimicrobial? (Multiple choice)
3. National guidelines
4. Hospital regulations
5. Departmental habits
6. Personal experience
7. Instructions from superiors
8. Pharmacist's suggestion
9. Others, please specify _________
10. When there is inconsistency between SAP practices and the guidelines, in your opinion, what is(are) the main cause? (Multiple choice)
11. The guideline does not consider patient-specific differences
12. The risk of postoperative infection is low when using drugs based on experience
13. Administering drugs according to the guideline may increase the risk of postoperative infection
14. Following the instructions of superiors
15. The guideline lacks sufficient evidence-based medical evidence
16. Strict adherence to the guideline
17. Others, please specify _________
